# Supplementary material for: Differences in cardiovascular disease mortality between northern and southern China under exposure to different temperatures: a systematic review
Source: PeerJ. 2024 Oct 30;12:e18355. doi: 10.7717/peerj.18355 (PMC11531265; doi:10.7717/peerj.18355)
Supplement: Table S1 [file peerj-12-18355-s003.pdf]

Table S1 Summary of the assessment of the quality of the evidence on extreme cold, extreme heat and extremely high DTR as a risk factor for CVD.

Reference		Extreme cold(n=13)		Extreme heat(n=12)		Extremely high DTR(n=8)
	rating	bias	rating	bias	rating	bias
Quality of the evidence assessment						
**i=Downgrade considerations**						
Risk of bias across studies	0	There is no substantial risk of bias across most studies.	0	There is no substantial risk of bias across most studies.	0	There is no substantial risk of bias across most studies.
Indirectness	0	CVD mortality was appropriate outcome, studies conducted in the population of interest, mostly direct measures of exposure	0	CVD mortality was appropriate outcome, studies conducted in the population of interest, mostly direct measures of exposure	0	CVD mortality was appropriate outcome, studies conducted in the population of interest, mostly direct measures of exposure
Inconsistency	0	Effect estimates likely to differ because of differences in study methods and not be driven by unexpected heterogeneity	0	Effect estimates likely to differ because of differences in study methods and not be driven by unexpected heterogeneity	0	Effect estimates likely to differ because of differences in study methods and not be driven by unexpected heterogeneity
Imprecision	-1	Three studies had wide confidence intervals.	-1	One study had wide confidence intervals.	-1	One study had wide confidence intervals.
Publication bias	-1	The evidence of publication bias was inspected visually in the egger's test.	-1	The evidence of publication bias was inspected visually in the egger's test.	0	No evidence for publication bias for studies that would meet our inclusion criteria.
**ii. Upgrade considerations**						
Size of the effect	1	Effect sizes are big in most studies	1	Effect sizes are big in most studies	1	Effect sizes are small in most studies
Dose response pattern	1	Most studies report broadly similar dose–response pattern	1	Most studies report broadly similar dose–response pattern	1	Most studies report broadly similar dose–response pattern
Confounding minimizes	0	There is no evidence to suggest that possible residual confounding factors reduce the estimation	0	There is no evidence to suggest that possible residual confounding factors reduce the estimation	0	There is no evidence to suggest that possible residual confounding factors reduce the estimation

effect		of effectiveness		of effectiveness		of effectiveness
**iii. Summary of the quality assessment**						
Overall quality of evidence starts: Moderate	Moderate	Moderate + (-1) + (-1) + 1 + 1 = moderate, Downgrading/upgrading resulted in moderate rating for the quality of evidence	Moderate	Moderate + (-1) + (-1) + 1 + 1 = moderate, Downgrading/upgrading resulted in moderate rating for the quality of evidence	High	Moderate + (-1) + 1 + 1 = high, Downgrading/upgrading resulted in high rating for the quality of evidence
Summary of findings	n/a	Overall, the evidence for a higher risk of cardiovascular mortality with extreme cold exposure was of moderate quality	n/a	Overall, the evidence for a higher risk of cardiovascular mortality with extreme heat exposure was of moderate quality	n/a	Overall, the evidence for a higher risk of cardiovascular mortality with extremely high DTR exposure was of moderate quality
Strength of evidence assessment						
Quality of evidence	Moderate		Moderate		High	
Direction of effect estimates	n/a	Direction largely as expected	n/a	Direction largely as expected	n/a	Direction largely as expected
Confidence in effect estimate	n/a	Studies on extreme cold measure directly the outcome of interest, direction of effect is largely consistent, majority score low on risk of bias.	n/a	Studies on extreme heat measure directly the outcome of interest, direction of effect is largely consistent, majority score low on risk of bias.	n/a	Studies on extremely high DTR measure directly the outcome of interest, direction of effect is largely consistent, majority score low on risk of bias.
Other aspects	n/a	Differences in contextual factors, including population exposure level and vulnerability, differences in physical and physiological adaptation across study populations make interpretation and comparison difficult.	n/a	Differences in contextual factors, including population exposure level and vulnerability, differences in physical and physiological adaptation across study populations make interpretation and comparison difficult.	n/a	Differences in contextual factors, including population exposure level and vulnerability, differences in physical and physiological adaptation across study populations make interpretation and comparison difficult.

Overall strength of evidence	sufficient	We found sufficient evidence that extreme cold is positively associated with cardiovascular disease mortality in China, in which case chance, bias, and confounding factors can be reasonably excluded. The available evidence includes the results of one or more well designed studies, and quantitative estimates can only be interpreted broadly because of the lack of comparability across studies.	sufficient	We found sufficient evidence that extremely heat is positively associated with cardiovascular disease mortality in China, in which case chance, bias, and confounding factors can be reasonably excluded. The available evidence includes the results of one or more well designed studies, and quantitative estimates can only be interpreted broadly because of the lack of comparability across studies.	sufficient	We found sufficient evidence that extremely high DTR is positively associated with cardiovascular disease mortality in China, in which case chance, bias, and confounding factors can be reasonably excluded. The available evidence includes the results of one or more well designed studies, and quantitative estimates can only be interpreted broadly because of the lack of comparability across studies.
